# New Jersey

**Published:** 1899-11

**Authors:** S. Lockwood

**Affiliations:** Secretary


Secretary.

### VETERINARY MEDICAL ASSOCIATION OF NEW JERSEY.

THE Association held its forty-fourth regular meeting in the Arlington Hotel, 495 Broad Street, Newark, on September 14, 1899, at 11 o'clock, the President in the chair. Eleven members were present. The minutes of the previous meeting were read and approved.

Dr. W. Herbert Lowe was unanimously elected a member of the Association. Dr. Lowe urged the co-operation of the members to secure the centralization of the veterinarians' interests in the State.

A committee of five was appointed to co-operate with the veterinarians of the State and urge the calling of a convention to discuss plans for the benefit of the profession in the State. The committee consisted of Drs. Miller, Runge, Lowe, Hasbrouck, and Kilburn. Adjourned at 3.30 to meet at the same place in January, 1900.

S. Lockwood,
Secretary.

### WORK AMONG BUREAU MEN.

As an incentive to more systematic and profitable study along the lines of work in which they are engaged, several of the employés of the Bureau of Animal Industry at Indianapolis, Ind., met quite recently and organized a local society. The officers elected were as follows: President, Dr. Tait Butler; Vice-President, Dr. T. L. Armstrong; Secretary-Treasurer, Dr. F. T. Dolan; Programme Committee, Dr. R. W. Tuck, Dr. S. G. Hendren and Martin Grady. The first regular meeting will be held on October 4th, for which an interesting programme has been prepared.

